# Athletes and Hypertension

**DOI:** 10.1007/s11886-021-01608-x

**Published:** 2021-10-16

**Authors:** Victor Schweiger, David Niederseer, Christian Schmied, Christine Attenhofer-Jost, Stefano Caselli

**Affiliations:** 1 Department of Cardiology, University Heart Center Zurich, University Hospital Zurich, Rämistrasse 100, 8091 Zurich, Switzerland; 2 Cardiovascular Center Zurich, Hirslanden Klinik im Park, Zurich, Switzerland

**Keywords:** Hypertension, Athletes, Blood pressure, Exercise, Power sports

## Abstract

**Purpose of Review:**

We reviewed most current medical literature in order to describe the epidemiology, clinical manifestation, outcome, and management of hypertension in athletes.

**Recent Findings:**

An estimated quarter of the world’s population is suffering from hypertension and this prevalence is also reflected in athletes and in individuals involved in leisure time sport activities. Several studies found an inverse relationship between physical activity and blood pressure. Therefore, physical exercise is recommended to prevent, manage, and treat hypertension. On the other hand, the prevalence of hypertension may vary by sport and in some cases may even be higher in athletes competing in certain disciplines than in the general population. Hypertension is the most common medical condition in athletes and may raise concerns about its management and the individual’s eligibility for competitive sports. A thorough clinical evaluation should be performed to correctly diagnose or rule out hypertension in athletes, describe the individual’s risk profile, rule out secondary causes, and detect possible hypertension-mediated organ damage caused by hypertension at an early stage. Based on most recent clinical research and international consensus documents, we propose a diagnostic algorithm as well the non-pharmacological and pharmacological management of hypertension in athletes.

**Summary:**

Although elevated blood pressure levels are less common in the active population, athletes are not protected from hypertension. A thorough diagnostic approach may help to identify individual at risk for adverse cardiovascular events and to address the optimal treatment as well as sport recommendations.

## Introduction

Hypertension affects approximately 25% of the world population [1••]. Not only is hypertension one of the most prevalent medical disorders but it is also associated with an increased cardiovascular morbidity and a higher all-cause mortality [2]. Epidemiological studies reported an inverse relationship between physical activity and blood pressure (BP) levels suggesting that both aerobic and resistance exercise lower the systolic and diastolic BP [3–5]. Therefore, the European Society of Cardiology (ESC) and the American Heart Association (AHA) recommend exercise for the prevention and therapy of hypertension [2].

The high prevalence of hypertension in the general population is also reflected in athletes, where it represents the most common condition identified at preparticipation screening and may potentially represent a reason for exclusion from competitive sports [2, 6]. This may seem counterintuitive since athletes should benefit from the positive cardiovascular effects of regular physical activity; however, it is also well known that high-intensity competitive exercise, in individuals with cardiovascular disease, may be associated with serious adverse events and increased risk of sudden cardiac death (SCD) [2, 7–10]. Specifically, arterial hypertension causes left ventricular hypertrophy on the long term and is a risk factor for coronary artery disease; in addition, an acute increase in blood pressure can destabilize atherosclerotic plaques and cause myocardial infarction or cerebrovascular events [1••].

In this review, we discuss the epidemiology, definition, clinical presentation, and outcome associated with hypertension in athletes, as well as preparticipation evaluation and management.

## Epidemiology of Hypertension in Athletes

In the largest hypertension study conducted on European athletes, Caselli et al. reported a prevalence of hypertension of 3% among a large cohort of competitive athletes (*n* = 2040, 64% men). In a large-scale (*n* = 138,390) systematic review, Berge et al. reported a prevalence of hypertension among athletes similar to that of the sedentary population but found that BP levels vary greatly between athletes participating in different kinds of sports [2]. Power sports in particular seemed to be associated with higher systemic BP levels [2]. As a matter of fact, several studies reported that athletes participating in weightlifting, rowing, and American-style football have higher BP levels and are more likely to suffer from prehypertension or hypertension compared to endurance athletes, with a prevalence of hypertension ranging from 8.8 to as much as 25.6% [2–12]. The generally higher body mass index (BMI) among strength athletes and football players in particular, as well as the chronic abuse of illicit drugs, supplements, or NSAIDs (non-steroidal anti-inflammatory drugs), could account for the increased prevalence of hypertension in these athletes [7, 8, 13, 14].

Hypertension also primarily occurs in male athletes [2] and BP levels exhibit a linear correlation with BMI, height, and the amount of training per week [2, 8, 15]. Furthermore, risk factors include diabetes mellitus, smoking, dyslipidemia, abdominal obesity as well as a positive family history of early onset cardiovascular disease [16]. A gender-specific risk factor for hypertension is the use of oral contraceptives. A study demonstrated that about 5% of women taking oral contraceptives develop hypertension over the course of 5 years [17].

## Definitions and Patterns of Hypertension in Athletes

Table 1 displays the classification of hypertension according to the European Society of Cardiology. Regarding the clinical presentation, several patterns have been observed in athletes and they, in part, differ from what is seen in the non-athletic normal population.

**Table 1:** Recommended values as stated by the ESC, while the ACC/AHA guidelines stages values > 130 as hypertension

**Classification**	**Systolic (mm Hg)**	**Diastolic (mm Hg)**
Optimal	<120	And < 80
Normal	120–129	Or 80–84/and < 80
High normal	130–139	Or 85–89/and 80–89
Stage 1 hypertension	140–159	Or 90–99
Stage 2 hypertension	≥160	Or ≥ 100
Isolated systolic hypertension	≥140	And < 80

Adapted from 2018 ESC Hypertension Guidelines

### Sustained Systemic Hypertension

Sustained systemic hypertension is defined as a systolic blood pressure > 139 mm Hg and/or diastolic blood pressure > 89 mm Hg measured on multiple occasions. It is a relevant and well-known independent risk factor for cardiovascular disease (CVD) [1••, 20].

### White Coat Hypertension

White coat hypertension (WCH) is defined as an elevated in-office BP, but normal BP during ambulatory monitoring [14]. Evidence about the prevalence of WCH in athletes is scarce. In one study on 410 male adolescent athletes, 16 out of 18 patients who presented with elevated office BP levels had a normal ambulatory BP measurement (ABPM) (24-h average, daytime and nocturnal), underlining the importance of the routine use of ABPM [18]. However, despite the low or normal overall BP levels, the ESC clearly states that there is a prominent association between WCH hypertension and an increased prevalence of dysmetabolic risk, as well as asymptomatic organ damage and an overall increased risk of CV events [14].

### Masked Hypertension

Masked hypertension (MH) is defined as normal office BP levels with elevated BP levels at ABPM (> 135/85 mm Hg out of-office and < 140/90 mm Hg in-office), and is also associated with an increased prevalence of dysmetabolic risk and asymptomatic organ damage [14]. Furthermore, long-term risk of fatal and non-fatal CV events is similar to that of patients with sustained hypertension [14]. MH has a higher prevalence in younger patients. One study reported a prevalence of 38% in a cohort of middle-aged endurance athletes and another study reported a prevalence of 35% in a collective of professional soccer players, indicative of a possibly high occurrence of MH among athletes participating in endurance and mixed dynamic sports [19, 20].

### Hypertensive Response to Exercise

Hypertensive response to exercise (HRE) is defined as a difference between peak and baseline systolic BP of at least 60 mm Hg in men and at least 50 mm Hg in women during exercise testing, or as a systolic BP exceeding 210 mm Hg in men and > 190 mm Hg in women [21, 22, 23•]. A large study on consecutive Italian Olympic Athletes examining blood pressure adaptation to exercise stated that the upper normal values of blood pressure during maximal exercise testing are 220/85 mmHg in male and 200/80 in female athletes [26]. Notably, HRE is associated with an increased incidence of hypertension at long-term follow-up, particularly if the patient experiences the increase during moderate-intensity exercise [23•]. Furthermore, HRE seems to be associated with a higher degree of cardiac remodeling [24]. In this context, it is important to note that, during weightlifting, BP can even temporarily rise to levels of as much as 480/350 mmHg [25].

### Isolated Systolic Hypertension

Isolated systolic hypertension (ISH) is a condition commonly found in the young and might reflect the most common hypertensive subtype among adolescents and adults [30, 31]. In the past, the condition was often considered benign. However, a recently published review by McEniery et al. questioned the benign nature of this condition [32]. They demonstrated that the individuals with ISH, in addition to the higher differences between brachial and central BP, also had higher central BP levels in general, increasing the risk for sustained hypertension later in life. They further stated that a central BP over the threshold of 120 mm Hg increases the incidence of sustained hypertension later in life (OR = 6.2). In a systematic review, Hokyou et al. demonstrated that ISH was associated with an increased CV risk, compared to the general population [33]. Notably, individuals suffering from stage 2 ISH were at higher risk of CV events compared to individuals with stage 1 systolic and diastolic hypertension.

## Long-Term Outcome in Professional Athletes

Several studies have shown that athletes in general experience a decreased mortality, and that endurance athletes in particular benefit from an increased longevity. Common examples from this category include cross-country skiers and runners [26–30]. Interestingly, the most prominent decreases are observed at low to moderate levels of exercise. These findings must be seen in the context of a generally healthier lifestyle led by athletes compared to the sedentary population, exemplified by much lower rates of unhealthy habits, such as smoking, diet-related factors, or sedentary behavior [31–33].

However, this might not apply to all kinds of athletes, as a study by Runacres et al. revealed that athletes participating in power sports do not reap the same benefits commonly experienced by endurance athletes, i.e., the all-cause and CVD mortality were not significantly different from those of the general population [34]. In another study, power sport participation was even associated with an increased risk of CVD compared to this population [35]. In fact, the majority of small studies actually seem to point towards an increased mortality in certain power sports (such as American football) compared to other sport disciplines [36–39].

It is reasonable to hypothesize that the increased mortality could, at least in part, be a consequence of the elevated BP levels among power sports athletes, as every BP increase by 20 mmHg over 115 mm Hg doubles the cardiovascular mortality [40]. Namely, hypertension promotes the occurrence of atherosclerotic plaques in peripheral vessels and in the coronary arteries [41]. Increased intima thickness and arterial stiffness, both commonly found in weightlifters, have already been found to increase mortality in the sedentary population [42, 43]. The colossal importance of controlling BP levels is perhaps best illustrated by a study that reported a 5% decrease in cardiovascular mortality in response to a very minimal reduction of 3 mm Hg SBP and DBP [44].

## Preparticipation Examination

Preparticipation evaluation (PPE) of athletes was introduced nearly 40 years ago with the aim of identifying athletes with cardiovascular diseases and potentially increased risk for SCD. The rationale for screening the athletes is the 2.5 higher incidence of SCD among these individuals compared to the normal population, which in most cases is related to an identifiable structural or electrical cardiac disorder [8, 11, 20, 24, 45–47]. The current ESC guidelines on sports cardiology and exercise in patients with cardiovascular disease recommend different screening regimes depending on national regulations and age [48••]. The Italian PPE of Olympic athletes consisting of clinical history, physical examination, BP measurement, ECG, exercise testing, and echocardiography represents an excellent example [49, 50].

**Fig. 1:**
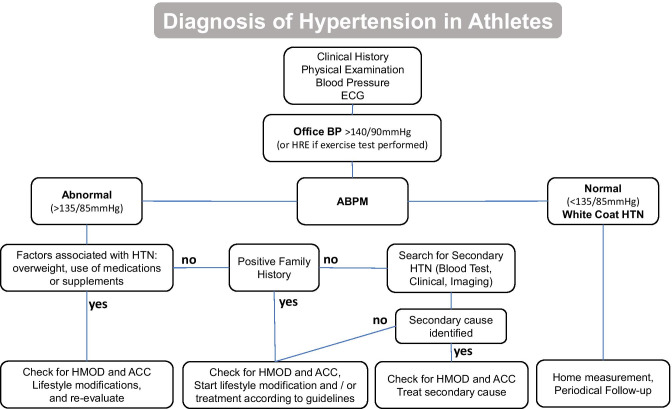
Preparticipation evaluation of athletes with hypertension (HTN). The first steps include family history, clinical examination, BP measurements, and ECG. In case the blood pressure (BP) is elevated when measured in the office, ambulatory BP measurement (ABPM) should be suggested, and further testing should be performed based on the results. HMOD, hypertension-mediated organ damage; ACC, associated clinical conditions

In Fig. 1, we present a diagnostic flowchart for athletes with suspected arterial hypertension. The initial evaluation consists of clinical history, physical examination, blood pressure measurement, and resting ECG. Blood pressure measurements should be carried out as recommended by the ESC [14]. However, it should be noted that the selection of an appropriate arm cuff size is of particular interest in this population, e.g., because of the larger upper arm circumference of strength athletes compared with the normal population, which may lead to biased higher blood pressure measurement results [51]. If office blood pressure values are > 140/90 mmHg, or if HRE occurs on exercise testing, ABPM is recommended to correctly diagnose patients with WCH. In case ABPM confirms hypertension, further diagnostic may be required based on family history or clinical examination. Appropriate sex- and age-adjusted percentile tables should be used when screening young athletes to properly identify hypertension in these individuals. According to the ESC, the threshold for hypertension is at the 95th percentile, whereas results between the 90th and 95th percentile only require appropriate follow-up measurements [52]. If hypertension is confirmed by APBM, further diagnostic measures may be required based on family history or clinical examination. Both the European Association of Preventive Cardiology and the Bethesda Conference suggest that diagnostic testing, such as limited laboratory testing for secondary causes of hypertension, should be performed under certain circumstances, as these may account for 5–10% of diagnosed HTN in athletes [53, 54]. Signs of secondary hypertension include an onset under the age of 30, the absence of risk factors including a positive family history, stage 3 hypertension, and a suddenly occurring stage 2 hypertension or resistant hypertension [1••].

Additional echocardiography should complement the PPE in athletes diagnosed with hypertension to detect signs of hypertensive heart disease. However, this can prove difficult in athletes as the left ventricle tends to become hypertrophied from intense exercise. Echocardiographic clues that may indicate hypertensive heart disease include [1••] the following:

1. Increase in wall thickness and mass beyond what would be expected based on age, gender, race, and type of sport (as an example, 12-mm septal thickness in a white male professional golfer may not be considered a typical adaptation to training)
2. Concentric hypertrophy of the left ventricle; this means an increased left ventricular mass with respect to ventricular size
3. Diastolic dysfunction on Doppler and Tissue Doppler echocardiography
4. Other signs may include a left atrial dilatation, enlargement of the thoracic aorta, or impaired left ventricular deformation on strain analysis

A maximal exercise test should be performed in all athletes with arterial hypertension in order to exclude coronary artery disease or exercise induced arrhythmias [1••, 48••].

In master athletes (> 35 years) with very high cardiovascular risk profile, a coronary computed tomography angiography (CCTA) may also be taken in consideration to search for coronary artery calcifications or stenosis [55].

Finally, other hypertension-mediated organ damage (HMOD) or associated clinical conditions (ACCs) should be sought-after, as these may provide additive relevant prognostic information (Table 2) [1••]. Furthermore, annual follow-ups should be conducted to assess possible reversal or progression of HMOD [1••]. The annual assessments should include resting and exercise ECG, echocardiography, kidney function testing, and/or retinal examination [1••].

**Table 2:** HMODs and associated clinical conditions

**HMODs**
LV hypertrophy (not considered as athletes heart)
Diastolic dysfunction
Ultrasound evidence of arterial wall thickening or atherosclerotic plaque
Hypertensive retinopathy
Increase in serum creatinine (1.3–1.5 mg/dL in men or, 1.2–1.4 mg/dL in women) and/or microalbuminuria
**Associated clinical conditions**
Atrial fibrillation
Heart failure
CVD (cerebrovascular disease, peripheral artery disease, or coronary artery disease)
Advanced retinopathy
Renal impairment

Adapted from 2018 ESC Hypertension Guidelines and the EAPC recommendations for participation in competitive sports of athletes with arterial hypertension

## Sport Eligibility in Athletes with Hypertension

The benefits of exercise far outweigh the potential risks and should therefore be integrated into every patient’s daily routine. The WHO modified their recommendations to 150–300 min of moderate-intensity aerobic physical activity and additional muscle-strengthening training on 2 or more days a week, increasing the requirements set out by the previous set of guidelines [56].

The EAPC recommends that participation for athletes with high to moderate cardiovascular risk should not be restricted if the BP levels are lower than the respective hypertensive threshold [16, 57]. However, if BP levels exceed 139/89 mm Hg, lifestyle changes or immediate medical therapy (in case of stage 3 hypertension) should be introduced in order to help normalize BP levels. Further recommendations depend on the presence of HMOD or ACCs. Without ACC or HMOD, athletes with previously diagnosed hypertension should be able to compete in all types of sports, so long as BP levels are normal. However, re-evaluation should be performed at regular intervals. If either ACC, HMOD, or both are present, all further risk factors should be controlled and, if possible, adjusted before granting the athlete the right to participate again. However, this recommendation is different for power sports, which should be avoided in case of hypertension and HMOD or ACC, even if the BP is well controlled [1••].

In the past, the 36th Bethesda Conference expressed a less strict view on the matter [58]. It stated that, in athletes with BP levels exceeding 139/89 mm Hg, an echocardiography should be conducted, and only if left ventricular hypertrophy beyond what is seen as reconcilable with an “athlete’s heart” is detected, sports participation should be restricted until further BP normalization can be achieved using appropriate pharmacological therapy.

The American Heart Association stated that stage 1 hypertension in the absence of target-organ damage should not limit eligibility for any competitive sport [59]. ABPM should be performed in all athletes with initially elevated hypertensive blood pressure measurements. Nevertheless, athletes suffering from stage 2 hypertension should be restricted from high static sports as weightlifting, boxing, and wrestling until hypertension is controlled. If hypertension is present parallel to another cardiovascular disease, eligibility for competitive sports participation should be based on type and severity of the associated condition.

The American college of sport medicine suggests that, in asymptomatic athletes without any risk factors or with 1 risk factor (not diabetes) but without HMOD/ACC, with hypertension stage 1 or even 2, who engage in light to moderate dynamic physical activity (intensity < 60% V̇O_2_R), there is generally no need for further testing beyond the routine evaluation [60]. However, all hypertensive patients who are planning to engage in high or very high-intensity exercise (intensity ≥ 60% V̇O_2_R) should, at the very least, undergo a medically supervised peak or symptom-limited exercise test with ECG monitoring.

## Treatment of Hypertensive Athletes

**Fig. 2:**
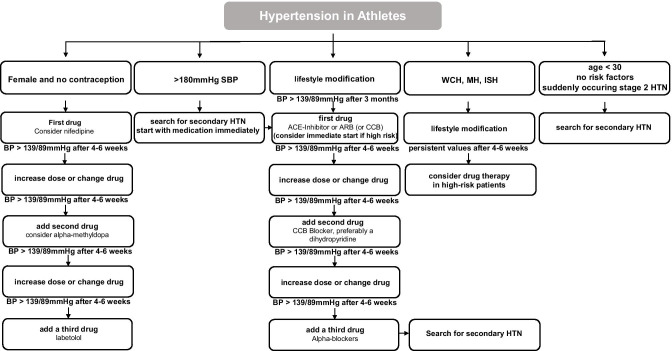
Possible treatment scheme for athletes with hypertension. This flowchart provides a possible treatment regimen for athletes with hypertension who are resistant to lifestyle modifications. Based on the initial findings, secondary causes of hypertension should be sought or direct therapy should be initiated. Abbreviations: SBP, systolic blood pressure; WCH, white coat hypertension; ISH, isolated systolic hypertension; HTN, hypertension; ACE angiotensin-converting enzyme; ARB, angiotensin receptor blocker; CCB, calcium channel blocker

Athletes with hypertension should be treated according to the general ESC guidelines for hypertension [1••, 14]. Figure 2 shows our suggested adapted protocol for athletes. A first step should always include non-pharmacological strategies like salt restriction, sufficient physical activity, weight reduction if obesity is present, alcohol restriction, strict nicotine cessation, healthier eating habits, and discontinuation of supplements, anti-inflammatory, or enhancing drugs [1••, 14]. In addition, high potassium intake and endurance exercising, if not already implemented in the training routine, might be beneficial [1••, 14, 61]. If the implemented lifestyle changes do not sufficiently lower the BP after 3 months, antihypertensive medications should be considered [1••, 14]. However, in some cases, such as newly diagnosed stage 3 hypertension or in individuals at high to very high risk of cardiovascular complications, drug therapy should be initiated immediately [1••].

Currently, there are no recommendations for the management of patients with WCH, ISH, MH, and HRE. However, as studies suggest that individuals suffering from these BP patterns are at an increased cardiovascular risk, lifestyle changes should be considered to lower the cardiovascular risk profile. Randomized controlled trials will be required to determine whether individuals suffering from one of those BP patterns could benefit from pharmacological therapies. In addition, follow-ups should be very thorough to identify individuals at high risk for CV events.

Diuretics are banned as doping agents under the World Anti-Doping Agency (WADA)-Code because of their potential to mask enhancing substances in the bloodstream during laboratory testing and may therefore be used in athletes only if a therapeutic use exemption (TUE) has been granted. Angiotensin-converting enzyme (ACE) inhibitors should be considered a first-line therapy [17, 62]. In patients who cannot tolerate ACE inhibitors, the use of an angiotensin II receptor antagonist (ARB) is recommended [17]. Both agents have been shown to have no significant effects on energy metabolism and do not impair the maximal oxygen uptake [63, 64]. In fact, a recent review by Wang et al. even suggests a possible performance-enhancing effect of ACE inhibitors, as lower plasma ACE levels in patient populations have been associated with improved muscle efficiency and increases in muscle mass and capacity [65].

Alternatively, or additionally, calcium channel blockers (CCB) may be considered a first-line therapy. Although CCB therapy can result in decreased cardiac contractility and thus compromise cardiac output, a study found that VO_2_ max and endurance performance were not affected by these agents [17, 66, 67]. There are two classes of CCBs, dihydropyridines and non-dihydropyridines. Dihydropyridines have been shown to maintain the hemodynamic profile as effectively as ACE inhibitors and may have a better hemodynamic profile compared to non-dihydropyridines [67]. Unfortunately, dihydropyridines in particular can cause reflex tachycardia, whereas non-dihydropyridines can cause bradycardia, decreased maximal heart rate, and a decrease in left ventricular contractility [40]. Therefore, the use of dihydropyridines (e.g., amlodipine, lercanidipine, nifedipine) should be preferred when considering CCBs.

Alpha blockers may be prescribed as a replacement or additional therapy. They reduce systemic vascular resistance without increasing heart rate or cardiac output and have been shown to lower systolic blood pressure immediately after running [17, 68]. However, a crossover study has shown that alpha blockers significantly decrease VO_2_ max and physical endurance performance in athletic men with mild hypertension, leading to the conclusion that alpha blockers should be used primarily as second-line therapy [68].

Beta-blockers are banned by the World Anti-Doping Agency (WADA) for athletes competing in various competitive skill sports, including archery and shooting [62]. Because of their potential to decrease cardiac output and aerobic exercise performance, beta-blockers may be considered a third-line therapy for non-skill sports athletes [69]. In addition, endurance athletes often exhibit significant bradycardia, which could be exacerbated by this class of drugs [70].

Most hypertensive patients require two antihypertensive substances [40]. Therefore, combination therapy may also be required in athletes to normalize blood pressure levels. Unfortunately, there currently exists limited information on the hemodynamic consequences and implications of drug combinations for hypertensive athletes. A combination of ACE inhibitors or ARB with CCB seems to be the optimal, if required [71]. Furthermore, if a third agent is required, alpha blockers could be considered [72].

Hypertension is generally less common in female athletes than in male athletes, but pharmacologic therapy may still be required in the former [2]. In this case, it is essential to consider the teratogenic effects of ACE inhibitors and to provide adequately information to female athletes in this regard [73]. Other therapeutic agents or treatment strategies should always be considered for female athletes planning pregnancy. First-line therapies with a dihydropyridine CCB, particularly nifedipine, or with alpha-methyldopa, both of which are also recommended as first-line in pregnancy, are nonteratogenic alternatives [74]. If necessary, beta-blockers, especially labetalol, may be considered second-line therapy in female athletes or added to combination therapy [74].

## The Role of Supplements on Blood Pressure Levels

As mentioned above, supplements as well as illicit substance abuse might be causative for the development of hypertension in some athletes. In this review, we emphasize only on some of the most widely used supplements and doping substances and their effect on blood pressure levels.

A study of American football players examined the effects of NSAID use on blood pressure and weight. Among this study population, daily users of NSAIDs had the highest blood pressure levels and weight. However, the fact that daily NSAIDs use was associated with increased weight may also indicate that there are more chronic users among linemen, who have been reported to be more prone to hypertension in general [75]. Nevertheless, a meta-analysis conducted by Snowden et al. found that NSAID use in the normal population is associated with an increase in blood pressure of 1.4 to 14 mmHg, with the highest levels in users already receiving therapy for hypertension [76].

One substance class commonly known to increase blood pressure is anabolic steroids. In a cross-sectional study published in 2018, Rasmussen et al. revealed that anabolic steroid abuse increases blood pressure by up to 8 mmHg and increases the prevalence of hypertension threefold compared with nonusers [77]. Moreover, chronic anabolic steroid abuse seems to increase the aortic stiffness [77].

Another illicit enhancing substance is erythropoietin (EPO). One study on 8 normotensive individuals demonstrated that the use of EPO significantly increases the resting systolic blood pressure (96.7 ± 3.8 vs. 102.0 ± 6.0) [78]. However, another study on EPO abuse in healthy adults indicated that blood pressure levels increase exclusively during exercise [79]. Overall, larger studies need to be conducted to accurately assess the blood pressure changes caused by EPO abuse, especially in athletes.

While the former agents are able to increase blood pressure, human growth hormone (hGH), which anecdotally is commonly used by bodybuilders, has been shown to lower blood pressure in several studies [80–82]. However, because studies of the hemodynamic effects of GH have not been conducted in athletes but mainly in individuals with GH insufficiency seeking to obtain physiological GH levels, blood pressure changes in the former may be difficult to predict.

Another supplement that should be mentioned is creatine monohydrate, as it is one of the best-studied supplements with well-documented benefits. However, despite anecdotal reports, creatine monohydrate does not increase the prevalence of hypertension [83–85].

Methylphenidate is another agent commonly used by athletes. Studies suggest that it has the potential to increase the blood pressure by up to 5 mmHg [86, 87]. But again, these effects have been observed in the normal population and might differ in the active population.

In conclusion, the abuse of illicit or legal substances can have consequences on resting BP levels; however, studies regarding the effects of these agents in the active population are lacking due to several factors, including the difficulty in finding a study population with known illicit use of GH, anabolic steroids, and EPO in competitive sports.

## Conclusion

Although elevated blood pressure levels are less common in the active population, athletes are not protected from hypertension. Studies suggest that the prevalence of hypertension may vary by sport and, moreover, appears to be even higher in athletes competing in certain disciplines than in the general population. A thorough diagnostic approach may help to identify individual at risk for adverse cardiovascular events and to address the optimal treatment as well as sport recommendations. The first-line strategy for the treatment of hypertension in athletes usually consist of non-pharmacological lifestyle modifications and only if these measures are insufficient, pharmacological treatment should be initiated. However, pharmacological antihypertensive therapy in athletes remains challenging, and the safety and hemodynamic effects of combination therapy in this very cohort require further investigation.
